# Development of a statewide network hub for screening, referral, and enrollment into food as medicine programs across Kentucky

**DOI:** 10.3389/fpubh.2024.1502858

**Published:** 2025-01-07

**Authors:** Christa Mayfield, Carolyn Lauckner, Joshua Bush, Ethan Cosson, Lauren Batey, Alison Gustafson

**Affiliations:** ^1^Department of Dietetics and Human Nutrition, College of Agriculture, Food and Environment, University of Kentucky, Lexington, KY, United States; ^2^Department of Behavioral Science, University of Kentucky College of Medicine, Lexington, KY, United States; ^3^Kentucky Injury Prevention and Research Center, College of Public Health, University of Kentucky, Lexington, KY, United States; ^4^College of Nursing, University of Kentucky, Lexington, KY, United States

**Keywords:** hypertension, gestational diabetes, type 2 diabetes, food insecurity, diet-sensitive health outcomes, food as medicine

## Abstract

Widespread recognition of food as medicine interventions’ role in reducing food insecurity and improving health outcomes has recently emerged. Several states have released In Lieu of Services, state-approved alternative services that may be offered by managed care organizations in place of covered benefits, or 1,115 Medicaid waivers, which may allow for expanded nutrition services to reduce food insecurity and improve health outcomes. However, there are significant gaps in understanding how to create a statewide system for delivering “healthcare by food” interventions. The University of Kentucky Food as Health Alliance first piloted the development of a statewide hub facilitating referral to, enrollment in, and evaluation of food as medicine programs across two healthcare providers (one urban and one rural). We then used a quasi experimental study design to examine effects on systolic and diastolic blood pressure in a target population of Medicaid eligible individuals aged 18–64 with high blood pressure and/or type 2 diabetes in rural and urban areas. Participant allocation was based on geographic location for each program arm with no control group. This feasibility case study aims to: (1) outline the development of a referral system between healthcare and food as medicine providers; (2) describe gaps in referral and enrollment; (3) summarize lessons learned from a statewide network as a blueprint for other states; and (4) present clinical outcomes across three food as medicine programs. Ninety-two referrals were received from UK HealthCare with 21 enrolled in medically tailored meals and 28 enrolled in a grocery prescription (53% enrollment rate). Thirty-two referrals were received from Appalachian Regional Healthcare with 26 enrolled in meal kits (81% enrollment rate). On average, the reduction in systolic blood pressure was 9.67 mmHg among medically tailored meals participants and 6.89 mmHg among grocery prescription participants. Creating a statewide system to address food insecurity and clinical outcomes requires key support from a host of stakeholders. Policy steps moving forward need to consider funding and infrastructure for screening, referral, enrollment and engagement hubs for improved health outcomes.

**Clinical trial registration:**

ClinicalTrials.gov, NCT06033664.

## Introduction

Food insecurity is linked to worse diet quality, increased rates of mental health problems, diabetes, and heart disease (1–4). Food insecurity is consistently more prevalent among households with a person living with cardiovascular disease (CVD) and type 2 diabetes (T2DM), and similarly, CVD and T2DM are also more prevalent in food-insecure households (2, 5–8). Across the United States, food insecurity rates have doubled between 2011 and 2017 (9). In addition, nutrition insecurity, defined as lack of consistent affordable access to nutritious foods and beverages that promote health are prevalent among food insecure populations (10–12). Kentucky faces the burden of some of the nation’s worst health outcomes for CVD and T2DM (13) and has one of the highest rates of food insecurity (14). The confluence of higher rates of CVD and T2DM in Kentucky coupled with higher rates of food insecurity creates an urgent need to identify effective processes for referring patients in clinics to community-based food resources to better promote patient engagement and improved health outcomes.

One such clinic-linked resource which provides compelling evidence is food as medicine programs (15). Three common food as medicine interventions for food insecurity and diet-sensitive chronic disease are grocery prescription programs, medically tailored meals (MTM), and meal kits. Meal kits, or nontailored meals, are lower cost, have pre-determined portion sizes, and do not require shopping but do require cooking and meal preparation (16). MTM, on the other hand, provide already prepared meals tailored to the dietary needs of the individual and can reduce transportation barriers as they are delivered directly to patients. MTM are an intensive intervention that may produce improvements in hypertension (HTN) by providing appropriate food based in the Dietary Approaches to Stop Hypertension (DASH) diet, with as few barriers to consumption as possible (17, 18). However, MTM are resource intensive and may not be suitable for individuals who prefer more control over their diet. Lastly, grocery prescription programs allow the patient greater choice in their food selection and can provide delivery of items to the home but require cooking knowledge and meal preparation resources. While there is growing interest and emerging research related to delivery of food as medicine, there is a significant gap in understanding how to develop an effective statewide system for screening, referral, enrollment, and engagement to actually deliver these programs for improved health outcomes.

Screening is the first step to identify potential patients that may be eligible for these nutrition programs. Starting in 2024, the Centers for Medicare and Medicaid Services have mandated screening for social determinants of health (SDoH), including food insecurity. However, many health care providers are not screening in the same manner or consistently across patient populations (19). In addition, some patients may not admit to needing assistance in the provider’s office because of stigma or being unsure if there is support for themselves and their family members (20). There is suggestive evidence that affirmative responses to SDoH questions may be more readily given when questions can be answered privately, in a manner removed from interpersonal interactions (21). Furthermore, there may be less stigma associated with answering these questions when conducted in private and in an easily accessible manner, such as via text message (20, 22). One recent study found success with online screening mechanisms for food insecurity and other related SDoH (19). In addition, automated screening may provide less burden for providers, reducing time constraints and disruption to intake processes and clinic flow and increasing the likelihood of consistent implementation of screening protocols. There is a lack of evidence and practical research examining how to develop and implement a cohesive screening, referral, and enrollment approach to meet the shifting policy changes across healthcare providers.

**Figure 1:**
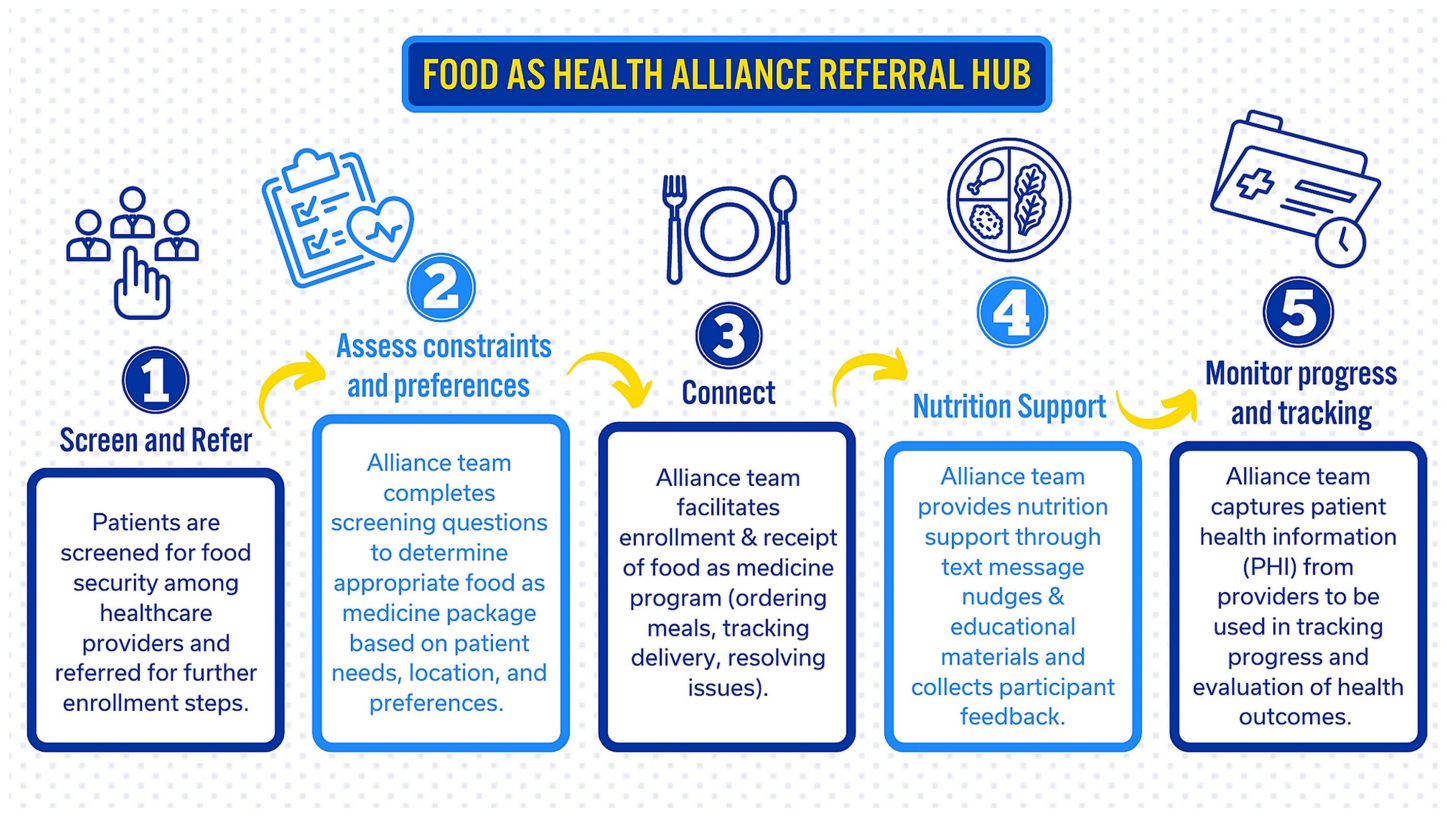
System for screening, referral, enrollment and engagement for food is medicine.

While there is energy toward addressing screening and referral, there is parallel work that needs to be done for food as medicine programs to be implemented in collaboration with health care providers (23, 24). There is a growing body of evidence that illustrates how partnerships between healthcare systems and local food assistance programs can improve dietary health (25, 26). Yet, there remain key barriers to implementing a statewide approach meeting diverse patient needs across disparate locations. Several states have encountered barriers related to inconsistent referral flow and bottlenecks that occur between healthcare providers, community partners offering the food programs, and patients (27). To relieve these pain points, the Food as Health Alliance launched in 2022 at the University of Kentucky to bring together clinical and community researchers spanning across agriculture, food, and healthcare sectors to address food insecurity and diet-sensitive chronic disease across Kentucky. With mandated SDoH screening in 2024, the Alliance began building capacity to pilot a hub for screening, referral, and enrollment between healthcare providers and food as medicine programs. Figure 1 is a conceptual model for the Alliance’s statewide food as medicine hub with screening, referral, enrollment, nutritional support, and monitoring and evaluation.

The Food as Health Alliance has begun building capacity among industry and community-based organizations to allow all residents in Kentucky to have access to a food as medicine program based on need and preferences. The development of these partners allows for the second part, which is testing the feasibility of various food as medicine programs (Meal Kits, Grocery Prescription, and MTM) to understand the scalability of the various programs. Offering diverse food as medicine programs allows a suite of options for patients rather than a one size fits all approach. The pilot testing of each food as medicine program can provide insight into how to enroll patients into each program and how individuals engage with these programs over time.

The goal of our pilot case study is to describe the feasibility, lessons learned, and pain points of developing key procedures for screening for food insecurity and referral of patients into a centralized hub for enrollment and engagement in a food as medicine intervention. Thus, our study aims to answer layered implementation science questions of how best to screen, refer, and enroll patients to address food insecurity and improve utilization in the short-term. In addition, these data points can then allow future research to test a fully powered study to determine dose, duration, and intensity of food as medicine to better meet patient needs while also improving clinical outcomes.

## Methods

### Context

This pilot engaged 2 geographically diverse hospital systems and 3 food as medicine providers to develop and test a system for screening, referral, enrollment, and engaging patients in a food as medicine program. This pilot was conducted at two large hospital systems, one in a metro area in Kentucky (UK Healthcare) and the other in a highly rural and remote area of Eastern, KY in the Appalachian region (Appalachian Regional Healthcare).

#### UK HealthCare

Based at the University of Kentucky, UK HealthCare includes a level 1 trauma center and over 80 specialized clinics and sees more than 642,000 ambulatory visits annually. For this pilot, one primary care clinic and one specialty clinic based in Fayette County, the second most populous county in Kentucky (28), were selected.

#### Appalachian Regional Healthcare (ARH)

Appalachian Regional Healthcare is the largest healthcare provider in central Appalachia. It operates 95 clinics in southeastern Kentucky and southern West Virginia with 827,073 outpatient visits annually. Two primary care clinics based in highly rural areas, Floyd County and Perry County (28), were selected for the program.

Table 1 shows the rates of poverty (29), unemployment (30), households receiving SNAP (31), and food insecurity by county (32) providing context for where the target healthcare facilities are situated and where patients were recruited and enrolled.

**Table 1:** Description of counties participating in statewide pilot.

County	Population	% below poverty level	% unemployed	% of households receiving SNAP	% food insecure
Fayette	322,570	14.9%	5.0%	8.4%	13.1%
Floyd	35,942	29.3%	6.8%	24.8%	22.8%
Perry	28,473	23.8%	7.5%	25.0%	21.4%

### Screening development

Screening methods differed across hospital systems. UK Healthcare began piloting an automated screening process in late 2023 whereby patients received the food security screening questions before their medical appointment. The screener was sent to them via their MyChart portal, the patient-facing platform associated with Epic electronic medical records. If the patient did not complete the screener before their appointment, the patient was provided a tablet at check-in at the provider office to complete. Conversely, ARH conducted the screening face-to-face among patients. The screener, in each location, utilized the validated Hunger Vital Sign™ screener (33), which asks the following two questions:

“Within the past 12 months, we worried whether our food would run out before we got money to buy more.”

“Within the past 12 months, the food we bought just did not last and we did not have money to get more.”

If the patient answered “often true” or “sometimes true” to either question, they were coded as at risk of food insecurity in the electronic health record.

### Referral

For this pilot, clinicians and staff from the selected clinics were first trained in how to screen and refer patients from the electronic health record into REDCap (34, 35), the HIPAA-compliant software used by the Alliance for referral management. Two trainings, one in-person and one virtually, took place with each clinic to review the location of the food security screener within the electronic medical record, the screening and referral protocols, and to provide REDCap training. In total, 15 clinic staff across 4 clinics were trained, requiring 5 h of Alliance staff time. Additionally, the Alliance established data sharing agreements for patient information with both hospital systems.

To submit referrals into the Alliance hub, clinic staff ran a weekly report to capture a list of patients at risk of being food insecure and who had a diagnosis in their chart of hypertension and/or type 2 diabetes. These individuals were contacted by clinic staff and asked if they wanted assistance with food. If the patient answered yes, their name, contact information, and pertinent medical information were uploaded into the Alliance’s REDCap system for enrollment into a food as medicine program. UK Healthcare has a dedicated Population Health team that responds to positive social need screens. While ARH uses the face-to-face screening and clinic nurses to submit referrals into the hub.

### Enrollment

Referred patients were eligible to enroll in the study if they were between the ages of 18–64, screened positive for risk of food insecurity, requested food assistance, had a current diagnosis of hypertension or type 2 diabetes, and were not unhoused. Eligible patients were sent a text message and email with a link to a brief description of the food as medicine program and an eConsent form via REDCap. After consenting, patients completed an online baseline survey consisting of demographics and the 10-item Household Food Security Survey Module (36). Following enrollment, patients were signed up for a food as medicine program depending on their location and program availability at the time of enrollment and were then sent information about next steps via email and mail.

For referred patients who did not respond to the initial text message, a second text message was sent two days later. If no response was received, Alliance staff, student workers, or volunteers made three attempts to reach the patient by phone to complete enrollment. Additionally, a contact phone number was provided in the enrollment text messages and email for patients who preferred enrolling via phone. Referral and enrollment into food as medicine programs occurred over a 2-month period for each hospital system during November 2023–March 2024. Ninety-two referrals were received, and 49 patients enrolled from UK HealthCare. Thirty-two referrals were received, and 26 patients enrolled from ARH.

### Intervention – dose

The three intervention arms were: (1) medically tailored meals, (2) grocery prescription, and (3) meal kits. Patients were assigned to an intervention arm based on geographic location and program availability.

1. *Medically tailored meals (MTM)*

Mom’s Meals® was the provider of medically tailored meals for patients enrolled in this arm of the pilot. The meals were based on either heart-healthy or diabetes-friendly dietary guidelines. Mom’s Meals is a well-established partner who currently provides meals via Value Added Benefits programs (37). Mom’s Meals has large scale and thus could reach any resident in Kentucky. The Alliance team submitted patient names and addresses, meal preferences, allergies, and any other restrictions to Mom’s Meals. Mom’s Meals then delivered meals each week to participants’ homes. For this pilot, Mom’s Meals were offered to Fayette County-based patients. Patients received 10 medically tailored meals delivered to their door per week for 12 weeks at a cost of $9.08 per meal. Twenty-one UK HealthCare patients were enrolled in MTM.

2. *Grocery prescription*

Instacart was the grocery prescription provider for patients enrolled in this arm of the project. Instacart has a new program titled “Fresh Funds” which allows a partner to create a healthy shopping platform with selected food items that meet dietary requirements for those with T2DM or HTN (38). The Alliance team with two registered dietitians (RD) created the Fresh Funds allowable foods list to comply with American Diabetes Association guidelines and American Heart Association guidelines. Eligible food items were then highlighted with a Fresh Funds tag on the online shopping page for participants in the program. The Alliance team assisted any patient that needed help with downloading the Instacart app and setting up their initial cart. Once a patient had their cart established online, the patient could repeat the same order the following month or add different items. Fresh Funds were offered to patients in Fayette County. Patients received $200 of Fresh Funds per month for 3 months to be used on approved items on Instacart. Twenty-eight UK HealthCare patients were enrolled in grocery prescription.

3. *Meal kits*

Meal kits that meet the American Diabetes Association guidelines were developed among a team of registered dietitians from Food City grocery store and the Alliance to test an intermediate offering relative to preference for cooking with nutritional support. The meal kits consisted of 4 meals meeting dietary guidelines along with recipes to prepare the food that came in each box for 8 weeks. Logistics and operations were a key part of capacity building to allow for the packaging, distribution, and delivery of meal kits to patients. The Alliance, Food City, and DoorDash met monthly for a year to develop program operations, including store selection, key operation and accounting protocols, management training, and DoorDash merchant portal training. Two weekly delivery windows were set to accommodate Food City store operations and ensure adequate DoorDash driver supply. Participants were able to select a preferred delivery window during enrollment. Meals kits were offered to patients in Eastern Kentucky, which is rural and geographically large with some patients residing 20 miles or more from the participating Food City. DoorDash has a standard delivery radius of 15 miles, thus a cost structure for incremental delivery mileage up to 20 miles was established with DoorDash. Patients received a meal kit valued at $50 with 4 recipes delivered to their door via DoorDash each week for 8 weeks. Thirty-two ARH patients were enrolled in meal kits.

### Intervention – nutrition and other support

1. All patients received bi-weekly text message related to food coping strategies, recipe videos,^1^ how to stretch your food dollars, and general nutrition information to manage diet-sensitive health conditions. Text messages were sent two times per week for 8-weeks. The content was derived from previous online shopping intervention testing and development (39). Example messages sent in week 2 of the program were:
  “Each week you’ll receive a variety of non-starchy green vegetables in your meal kits such as brussels sprouts and bell peppers. Non-starchy vegetables are high in fiber and help keep blood sugars low. Find more examples of non-starchy vegetables at https://foodashealthalliance.ca.uky.edu/plan-your-plate” “Can you set a goal this week to eat more green vegetables? Enter 0, 1, or 2 0 = no; 1 = maybe; 2 = yes.”
2. Patients could also opt-in to receiving a phone call with a registered dietitian to help with managing their condition or other support for grocery shopping.
3. Grocery Rx: Received reminders about using their funds before the end of the month. They also received links to recipe video demonstrations and behavioral nudges about how to put healthy food items in their cart.
4. Meal Kits: Received reminders about stretching their food dollar, how to store leftovers, links to recipe videos, and tips for how to purchase other healthy food items to complement what was provided in their meal kits.

### Patient health data, survey collection, and incentives

Patient health data was uploaded by clinic staff at two time points: baseline and post intervention. Biometric measures for height, weight, blood pressure, and HbA1c were collected. Survey data were collected from participants at baseline and post intervention related to demographics, food security status, participant satisfaction and feedback on program acceptability, suitability, and challenges, impact on budget, and purchases outside of the food as medicine program. Patients were coded as food secure, low food security, or very low security based on the number of affirmative responses to the 10-item Household Food Security Survey Module following the USDA Economic Research Service guidance. Patients received $25 for baseline data collection and $25 for post intervention data collection.

### Analysis

Descriptive statistics were conducted to compare means and percentages across the three study arms. ANOVA was used with Bonferroni correction to make comparisons within and between each study arms.

## Results

### Enrollment and participants

Ninety-two referrals were received from UK HealthCare. Of those, 21 were enrolled in MTM and 28 were enrolled in the grocery Rx program (53% enrollment rate). Thirty-two referrals were received by ARH, and 26 of those were enrolled to receive meal kits (81% enrollment rate). Forty-seven participants self-enrolled in response to our messages while 28 enrolled by phone. Program completion rates were 86% in MTM, 82% in the grocery Rx program, and 96% in meal kits.

Table 2 provides a description of participants across the three food as medicine program arms. On average participants were between 47–50 years of age. There were differences across the groups with regard to self-reported gender, a higher percentage of women enrolled in the MTM and Grocery Rx compared to the meal kits. In addition, the meal kit arm was based in Eastern, KY which is predominantly White, and thus, there were race/ethnicity differences between the meal kit and other two arms.

**Table 2:** Demographics – Medicaid adults 18–64 with HTN or T2DM food as medicine program comparison.

	Medically Tailored Meals (Mom’s Meals)	Grocery Rx (Instacart)	Meal Kits (Food City)
	Baseline (*n* = 21)	Baseline (*n* = 28)	Baseline (*n* = 26)
Age	50.10 (1.93)	47.21 (1.98)	50.6 (2.31)
Gender			
Male	14.29%	18%	54%
Female	85.71%	82%	46%
Race			
Black/African American	71%	71%	
White	24%	29%	100%
Asian	5%	0%	
Household income			
Less than $15,000	62%	54%	54%
$15,000–$29,000	29%	25%	19%
$30,000 >	10%	21%	27%
Education			
Less than HS	0%	4%	4%
Some HS (9th–12th grade)	38%	14%	12%
HS grad	29%	43%	50%
Trade or vocational school	5%	0%	4%
Some College	10%	21%	12%
College Degree	19%	18%	19%
BMI Mean	35.58 (2.52)	40.10 (2.17)	33.57 (1.67)
SNAP participation	52%	35%	32%
Food pantry use	32%	25%	22%
Food security			
High or Marginal	55%	37%	38%
Low	35%	42%	40%
Very low	10%	21%	22%

### Health outcomes

Table 3 indicates there were significant within-person changes in the MTM and Grocery Rx arms for systolic blood pressure. There were no significant changes within the meal kit arm of the study. Those receiving MTM had an average reduction of 9.67 mmHg systolic and those in the Grocery Rx had a 6.89 mmHg systolic reduction on average in blood pressure.

**Table 3:** Change between baseline and post intervention within each food as medicine program.

	MTM	Grocery Rx	Meal Kits
Systolic	−9.67 [1.34, 17.99]*	−6.89 [0.36, 13.43]*	−5.63 [−3.61, 14.86]
Diastolic	−1.38 [−3.56, 6.33]	−4.07 [−1.51, 9.66]	−4.29 [−1.35, 9.94]
HbA1C	−0.458 [−1.00, 0.08]	−0.129 [−0.10, 0.35]	0.29 [−0.41, 0.99]

^*^p < 0.05 significant change in health outcome within the food as medicine programs. There were no significant differences between study arms across the three food as medicine programs. Detecting differences across study arms was not the intent of the pilot, and thus the study was not powered to do so.

### Participant purchasing patterns and acceptability

Table 4 provides insight into how participants utilized the additional funds saved from receiving food as medicine programs across food categories and other household needs. Those in the MTM utilized the additional income supports (from highest to lowest) to purchase fruit, meat, vegetables, dairy, beverages, cooking items, snacks and pantry items. Those in the grocery Rx arm utilized their additional income supports to purchase pantry items followed by meats and dairy products. Those in the meal kit arm had a similar purchasing pattern as the MTM. All arms reported using the influx of funds from food supports for bills, household supplies, food, medications, and leisure activities. Table 4 also details participant feedback on and satisfaction with the program. Across all three arms, more diversity of food options was requested and there were issues with delivery. Those in the Grocery Rx and Meal Kit arms were most willing to continue participation if the program was not free. Participant satisfaction was high across all three arms, with the lowest satisfaction being from the MTM participants relative to the other two arms.

**Table 4:** Participant self-reported feedback on food as medicine programs.

Purchasing patterns on food outside of the food as medicine program	MTM	Grocery Rx	Meal kits
Snacks	29%	12%	27%
Meats	43%	16%	35%
Dairy	35%	12%	42%
Beverages	35%	16%	58%
Baked Goods	20%	2%	23%
Sweets	12%	8%	12%
Pantry	27%	22%	38%
Cooking Items	31%	18%	58%
Fruits	51%		46%
Vegetables	39%		42%
How Influx of funds from food supports supported other financial constraints			
Bills (+ gas bills)	✓	✓	✓
Household supplies (cleaning products, personal items, etc.)	✓	✓	✓
Extra food	✓	✓	✓
Medication	✓	✓	✓
Leisure activities	✓	✓	✓
Participant reported issues			
Lack of options (+ more options)	24%	14%	12%
Delivery issues/challenges (+ challenges with recipes)	5%	11%	12%
Short duration	0%	4%	0%
Participant reported strengths			
Delivery	24%	4%	15%
Types of items included (+ ease of preparing them)	10%	25%	19%
Helpful/nice staff	5%	14%	0%
Participant satisfaction			
Would participant recommend to a friend (yes)	84%	86%	91%
Would participant continue with program if it was paid for (yes)	68%	67%	73%
Would participant continue with program if the program wasn’t free (yes)	44%	67%	72%

## Discussion

This case study documents the Food as Health Alliance’s pilot to develop a statewide hub for screening, referral and enrollment between 2 geographically diverse healthcare systems and 3 food as medicine programs and engage 75 patients in a food as medicine intervention. Keys steps for developing a system approach to Healthcare by Food™ (40) include adequate buy-in and training for clinic staff to identify eligible patients, clear procedures for screening and referring eligible patients to a centralized hub, and adequate personnel time to enroll and maintain patient engagement throughout. Diverse food as medicine program offerings provide more tailored interventions addressing patient needs and preferences and may increase patient satisfaction along with promising impacts on health. Continued success of a statewide food as medicine system will require support from key stakeholders and policies for funding and infrastructure for a screening, referral, enrollment, and engagement hub to improve food insecurity and health outcomes.

There are several key factors contributing to the success of our pilot, as well as opportunities for improvements in the hub development. The first step in building a Healthcare by Food™ system is the screening process. In our pilot, there was variance in method of screening across health care providers, consistency of clinic staff buy-in, and clinic capacity to respond to SDoH screening. Some clinic sites utilized an automated screening procedure while others utilized a face-to-face process to screen for food security among patients. Our case study cannot elucidate how the screening variance may have impacted response rates, but our process highlights that having clinic buy-in and support is critical for identifying patients at risk of food insecurity and in need of assistance. Several other programs have suggested that streamlined and automated screening and coding within the clinical workflow (41), integrated systems for closed loop referrals (42), and additional staff training (43) can improve the screening process. These differences point to additional work needed to identify effective levers for improving screening consistency across different hospital systems and even clinics to ensure equitable access.

The second step in a statewide Healthcare by Food™ system is the referral process between the health care provider and the hub. We engaged two large hospital systems in making referrals, but referral numbers were low despite multiple training sessions. The Alliance received most referrals from UK HealthCare’s Population Health team, tasked with addressing patient social needs, and ARH’s diabetes educator, who provided these on top of their normal workloads. Manually submitting referrals outside of the clinical workflow may have been a barrier for some clinics referring more patients. Some studies have had success with automatic referrals from the electronic health record (44). Integration between REDCap and electronic health records was not available for this pilot. Our experience suggests that training alone is not sufficient to elicit referrals, and significant referral numbers may not be possible without automation or dedicated personnel. The Alliance is engaging relevant stakeholders in discussions on automated referral opportunities. Additionally, the Alliance has begun gathering information on providers’ user experience with screening and referral to identify key pain points and areas to improve.

The final component in our hub development was the actual enrollment into a food as medicine program. Our study had success with automated texts and emails inviting patients to enroll, addressing delays between screening and resource connection noted by others (45). In line with other findings (45), our work suggests that technology, particularly text messaging, can facilitate enrollment into food as medicine programs. Despite the benefits of technology-based solutions, multiple pathways to enrollment were needed to not exacerbate disparities. Enrollment rates were greatly increased by Alliance personnel making multiple follow-ups to facilitate connection to nutrition services.

Participant satisfaction was high across all programs, with the lowest satisfaction among those in MTM. Our results suggest a preference among some for choosing and cooking their own food. Those in the Grocery Rx and Meal Kits were more satisfied with the items included and more willing to pay for the program. Additionally, outside food purchases by Grocery Rx users were lower across all categories compared to other programs. More choice may have allowed Grocery Rx participants to more readily utilize the program to meet their overall food needs whereas those in MTM and Meal Kits needed to make more purchases on top of the food provided. These results in combination may highlight the need for a range of food as medicine offerings.

Results from participation in the programs highlight potential success of diverse food as medicine programs in improving health outcomes, providing exogenous funds to support purchasing of other needs, and strong patient satisfaction. While we were not powered to detect differences between and within the various programs, our results suggest that a MTM or Grocery Rx program can have positive effects on blood pressure. Relative to other studies (46), our results are similar in reducing systolic blood pressure among food insecure adults. Our results expand on previous programs to provide insight into how individuals engage with this program and highlight the patient perspective. Key components of our programs included automated and timely contact with referred patients, food delivery with all programs, Grocery Rx with choice, and capacity for a variety of program offerings for differing patient needs and preferences. Key barriers included clinics manually submitting referrals and low referral numbers, a lack of feedback loops, delivery challenges in remote areas of Eastern KY with unreliable cellular service, online Grocery Rx being inaccessible for patients without available cash balance, and high personnel time required to assist patients and monitor implementation.

Our program is situated within a body of work aiming to inform key policy recommendations from Centers for Medicare and Medicaid as well as from the White House. Recent advances in several states have included food and nutrition services as part of ILOS or 1,115 Medicaid waivers. Results from these studies have indicated that there are gaps in the screening, referral, and enrollment steps (24). In addition, there is a host of research gaps that need to be filled (26) related to dose, duration, and intensity to better inform at what level these services should and can be reimbursed. Our work points to the role that tailored food programs based on personalized needs can have, while also considering how to develop a system wide approach rather than a piece-meal approach.

One limitation of this study is that we did not have screening rates from clinics to further explore barriers in the screening process. Another limitation is that Meal Kits offered 4 less weeks than other programs due to store constraints, possibly contributing to the lack of observed change in systolic blood pressure relative to other programs. Our study did not allow for comparisons across program arms or household characteristics (rural vs. urban, household size, SNAP participation, etc.) as samples were small and treatments were not matched. Larger, randomized control trials are needed to elucidate the clinical effectiveness of MTM vs. Grocery Rx vs. Meal Kits (26). In addition, there can be unintended consequences of participating in these programs which rely on internet access which may have limited program access and use among some participants. These programs also may have inadvertently left out individuals that felt shame or embarrassment answering food security status questions.

### Lessons learned

Food as medicine interventions cannot be a one-size-fits-all approach, and program implementation requires flexibility (19, 23). Abilities and preferences (shopping and cooking independently vs. prepared meals, comfort with technology), barriers (transportation, internet access), and intervention intensity needed for disease management can vary according to individual circumstances. As such, the Alliance is building capacity with a variety of food as medicine providers. During this pilot, we were not yet able to offer choice for which program a patient received or to offer programs equally across healthcare systems. Our team encountered participants refusing to enroll or dropping out due to misaligned program features and participant needs. More research is needed to understand how to effectively design treatments for user needs without sacrificing clinical effectiveness in the long-term (47) and how this tailoring can influence patient utilization. In future efforts, the Alliance will test assessing barriers and ‘matching’ participants to a food as medicine program during enrollment. As more opportunities for food as medicine to be a medically covered benefit become available, similar work may be increasingly important as it can inform how to maximize impact on health in the most cost-effective manner.

Additional steps are needed for our statewide referral hub to be scalable and integrated into the healthcare system. With Centers for Medicare and Medicaid Services approving Medicaid 1,115 waivers and ILOS plans for nutrition services addressing health related social needs, a funding mechanism is required to support this statewide food as medicine infrastructure similar to other states (26, 48). Capacity building is still needed around feedback loops into electronic health records, integration with Medicaid for billable services, and a technology platform to manage all steps. The Alliance is also gathering patient perspectives on each step (screening, referral, enrollment, and program delivery) for a user-centered design approach. Opportunities exist for more community-based organizations (CBOs) engaged in food as medicine to coordinate referrals and program delivery across Kentucky. CBOs often have deep relationships and knowledge of their communities and can sometimes be more adaptable to specific, local needs. For example, Eastern KY has a specific need for stronger delivery networks. CBOs embedded in the community may be best positioned to identify a solution.

Given our preliminary success with piloting a statewide screening and referral hub, the Alliance is well positioned to collaborate with a variety of multifaceted stakeholders, including patients, healthcare, payers, government, CBOs, industry, and research, to advance the integration of nutrition services and healthcare across Kentucky.

## Conclusion

The University of Kentucky Food as Health Alliance highlighted the key steps needed for a statewide approach to food as medicine. Training and buy-in from clinicians are critical first steps in identifying eligible patients. Extensive personnel time is required for referral and enrollment to maintain patient engagement throughout. Various food providers are needed to ensure that food programs meet the needs and preferences of enrolled patients. Lastly, there needs to be a system approach for feedback loops between all parties involved for improved patient care. This extensive network can be successful at improving health outcomes with key financial and legislative support. Creating a statewide system to address food insecurity and clinical outcomes requires key support from a host of stakeholders. Policy steps moving forward need to consider funding and infrastructure for screening, referral, enrollment and engagement “food as medicine” hubs for improved health outcomes.

## Data Availability

The raw data supporting the conclusions of this article will be made available by the authors, without undue reservation.

## Footnotes

^1^ https://foodashealthalliance.ca.uky.edu/recipes
